# Unexpected medical undergraduate simulation training (UMUST): can unexpected medical simulation scenarios help prepare medical students for the transition to foundation year doctor?

**DOI:** 10.1186/s12909-016-0629-x

**Published:** 2016-04-14

**Authors:** Simon Watmough, Helen Box, Nick Bennett, Alison Stewart, Michael Farrell

**Affiliations:** Lecturer, Institute of Learning and Teaching, University of Liverpool, Cedar house, Liverpool, L69 3GE UK; Director of Clinical Skills & Simulation, Senior Lecturer, University of Liverpool, Liverpool, L69 3GE UK; Head of Clinical Education, St Helens and Knowsley Teaching Hospital NHS Trust, Whiston, Liverpool, L35 5DR UK; Head of Education & Learning, Aintree University Hospital NHS Trust, Liverpool, L9 7AL UK; Undergraduate Education and Quality Manager, Blackpool Teaching Hospitals NHS Foundation Trust, Whinney Heys Road, Blackpool, FY3 8NR UK

**Keywords:** Utilising simulation, Clinical skills, Preparedness for practice

## Abstract

**Background:**

Preparing medical students with the skills necessary to deal with emergency situations as junior doctors can be challenging due to the complexities of creating authentic ‘real life’ experiences in artificial environments. The following paper is an evaluation of the UMUST (Unexpected Medical Undergraduate Simulation Training) project; a high-fidelity simulation based training programme designed to emulate the experience of dealing with medical emergencies for final year medical students preparing for practice as Foundation Year trainees.

**Methods:**

Final year medical students from Liverpool University who undertake their clinical placements at Blackpool Teaching Hospitals NHS Foundation Trust and St. Helens & Knowsley Teaching Hospitals NHS Trust were randomly allocated into groups and took part in a series of four unexpected simulation based scenarios. At the beginning of the week in which the scenarios ran, participants were issued with a hospital bleep which they carried with them during their placement. At an unknown time to them, the participants were bleeped to attend a simulated emergency scenario, and on arrival to the Clinical Skills and Simulation facility, members of the education team undertook a standardised simulation scenario. Each session was recorded on video which the participants subsequently watched as part of a debriefing process. An assessment tool was developed to gauge whether the participants made progress in their learning over the course of the four sessions.

Focus groups were held with the participants in order to evaluate their experience of the programme, and questionnaires were later distributed to all participants once they had begun working as a Foundation Year trainee. The questionnaires asked them how relevant UMUST was in preparing them for dealing with medical emergencies.

**Results:**

The questionnaires and the focus groups clearly showed that the doctors felt like UMUST was very valuable in preparing them to work as junior doctors. They had enjoyed taking part in UMUST and thought was a realistic and useful part of their undergraduate training.

**Conclusions:**

The feedback from the focus groups and the subsequent questionnaires clearly demonstrate that participants felt the UMUST programme helped to prepare them as junior doctors in terms of dealing with emergency situations.

## Background

Since 1993 the General Medical Council (GMC) has consistently called for final year medical students in the United Kingdom (UK) to experience as closely as possible what it is like to work as a junior doctor [1–3]. Initially this involved the recommendation that final year students shadow junior doctors to learn what the role entailed, which was then followed by ‘assistantships’ which the GMC describes as ‘a period during which a student acts as assistant to a junior doctor, with defined duties under appropriate supervision.’ [3]; but the need to obtain consent from patients remains an ongoing issue [4] and junior doctors continue to feel ill-prepared for acute and emergency care [5].

Simulation based education programmes, which obviate the need for gaining consent, therefore have an important role to play in better preparing medical students for dealing with medical emergencies as they undergo the transition to Foundation Year 1 (FY1) doctors. The following paper evaluates the effectiveness of one such programme: UMUST (Unexpected Medical Undergraduate Simulation Training).

### The UMUST programme

The UMUST programme was initially developed in 2009 at St. Helens & Knowsley Teaching Hospitals NHS Trust; but when a member of the project team moved to Blackpool Teaching Hospitals NHS Foundation Trust in 2011, the programme expanded across both sites and became a collaborative venture. Both hospitals are situated in North West of England and have close links with the University of Liverpool.

At the time of this evaluative study, the UMUST programme operated as follows:

Following an initial expression of interest, a total of 33 students across two sites volunteered to take part and were split into 8 groups. On the Monday morning of the week they were timetabled to attend a UMUST session, the students undertook their individual clinical rotations across the Trusts. Whilst the students knew in advance which week(s) they would take part in the programme, they were not informed of the exact time or date. Each student was required to carry a hospital pager which would be activated to initiate an unexpected emergency scenario. The activation of the pager was a signal for the student to attend the Clinical Skills and Simulation centre where the scenario was carried out.

The first student to arrive at the centre was presented with the scenario by a member of the clinical skills team who enacted the role a staff nurse on a ward giving handover of the patient, including a brief history. The student collated this information and commenced their investigations using the ABCDE framework [6, 7] – the standard procedure for assessing acutely unwell patients – and subsequently informed the other participants as they arrived. The scenario was approximately 15–20 min in duration before ending, either by the participants phoning for help from a senior colleague, or phoning for handover (the faculty staff would play the roles of all other ‘staff’ in the scenario).

The programme comprised of a series of four simulated scenarios undertaken throughout the course of the year. For authenticity, and to ensure optimum educational outcomes, it was imperative to implement scenarios relevant to the practice of junior doctors [5], and therefore each scenario was designed and developed following a series of focus groups with various stakeholders, including FY1 doctors.

In total, 20 FY1 doctors across both hospital sites attended two focus groups. They were asked which recent medical emergencies they had attended and which emergency scenarios they felt they would have benefitted from more experience of as undergraduates. Additionally, cohorts of final year medical students were asked what they were most concerned about in preparing for being on-call as an FY1 doctor; and the Resuscitation, Critical Care and governance teams from both hospitals were asked to identify those situations in clinical practice that were not managed as well as expected. The responses from each stakeholder group were triangulated and a subsequent analysis revealed there to be a number of common themes including hypovolemia dehydration, opiate overdose, COPD pneumonia, and sepsis. These themes formed the basis of the simulated scenarios used in the programme.

Each UMUST session was filmed and shown to the participants twice as part of a subsequent debrief session. The majority of learning occurred during this session as the students were encouraged to reflect upon and discuss – both individually and as a team – what they had done well in the scenario, how they could have performed differently, and how they might improve upon their practice in the future.

As well as obtaining feedback through the debriefing process, a formal assessment tool called the Objective Simulation Assessment Tool or OSAT [8] was developed which allowed each cohort of participants to be appraised by the education team in order to gauge whether their performance improved from scenarios one – four. OSAT is based on the standard ABCDE guide [6, 7] for dealing with emergency situations. The letters of the alphabet refer to the order in which students must assess and manage a patient: Airway, Breathing, Circulation, Disability and Exposure. Each of these stages has a number of specific objectives unique to them depending on the scenario involved: the hypervolemia dehydration scenario, for example, has 44 specific objectives distributed throughout the five stages of the ABCDE guide; and for each of these objectives the students are scored either twp (objective completed), one (objective attempted/incomplete), or zero (not attempted). If the group initiated a Breathing assessment before an Airway assessment they would score one as they would have deviated from the required process.

### UMUST and the university of Liverpool curriculum

The curriculum is detailed elsewhere [9] but at the time of this study University of Liverpool had an integrated problem-based learning (PBL) curriculum where students took their exams at the end of fourth year to allow the final year to act as an “apprentice year”. During final year rotations students were expected to practice all aspects of patient care from history taking and examination to treatment plans, practising clinical procedures and undertaking an Advanced Life Support (ALS) course in preparation for working as a FY1 doctor. UMUST took place in this final year.

## Methods

### Timetable

The timetable of the programme varied for each site: students at Blackpool Teaching Hospitals underwent the four UMUST sessions approximately every two months between September and May; and students at St Helens & Knowsley Teaching Hospitals underwent the four sessions over a seven week period alongside a single rotation.

### Ethics

Ethical approval was sought and gained from the University of Liverpool Committee on Research Ethics, the Research and Development Audit Manager at St. Helens & Knowsley Teaching Hospitals NHS Trust and the Research and Development Committee at Blackpool Teaching Hospitals NHS Foundation Trust. All participating students were issued with an information sheet that outlined the programme when they attended their general induction at the hospitals. They were required to provide informed written consent to take part in the focus groups.

### OSAT tool

The OSAT scoring tool was used by the faculty staff during the debriefing session. Following the session, the staff convened to reconcile their scores.

### Focus groups

Two focus groups at each hospital were arranged in May and June 2012. The aim of these sessions was to capture the participants’ feedback on the programme prior to their graduation. Each session was approximately 45 min in duration and was facilitated by SW to ensure minimal bias, as SW was neither a clinician nor a teacher on the course.

The questions for each focus group were identical and were decided on by the authors of this paper. The students were initially asked about their overall impression and experience of the programme with a series of subsequent questions including:

- What was it like holding the pager?
- Do you think the programme prepared you for FY1?
- How effective were the debriefing sessions?
- How relevant were the scenarios?
- How was the programme relevant to your final year curriculum?
- What did you enjoy about the programme?
- How could the programme be improved?

The focus groups were recorded and transcribed *verbatim* by SW and were subsequently analysed using the framework approach which involves clear stages of data analysis: familiarisation; identifying a thematic framework; indexing; charting; mapping and interpretation [10]. The tapes and transcripts were reviewed in the first instance for familiarisation and key themes were subsequently identified by examining issues which emerged pre and post the programme. Next, the data was coded and the text was indexed using descriptors alongside various passages in the transcriptions. The data was then charted alongside the appropriate part of the thematic framework and, finally, the charts were mapped to explore associations between the themes.

The results provided in this paper (see below) are summarised according to the participants’ answers to the questions about specific elements of the programme, and the key themes which emerged from those answers. As verification of qualitative analysis is always required, HB/NB read the full transcripts independently of SW to ensure validity. Due to the homogenous nature of the participating group, saturation of themes was reached relatively quickly [10], and the use of identical questions for all focus groups furthermore assisted in achieving saturation.

### Questionnaires

A questionnaire, which was developed from a previously published survey involving postgraduate anaesthetic trainees [11], was distributed to the participants of the programme subsequent to their graduation in order to ascertain their views on what the impact of UMUST had whilst they were working as junior doctors: it was distributed in February 2013, thereby ensuring that participants had undergone six months of training as a post-graduate and could therefore reflect on the relevance of the programme to their experience as an FY1 doctor. The questions used included asking participants:

- How many times they attended UMUST
- Whether they remembered the scenarios
- How relevant the scenarios were
- Whether the programme had changed their approach to clinical practice
- Whether the programme had helped to prepare them for working as an FY1 doctor
- How the programme could be improved

### Data analysis

The data from the questionnaires was subsequently inputted to an SPSS for Windows spread sheet for analysis. Free text comments were analysed thematically [12].

## Results

### OSAT results

The data presented in Table 1 represents the total score each group generated following the completion of each scenario. Using the specific objective for each scenario the maximum score which could be obtained was identified. The maximum score was not a reflection of the difficulty of the scenario; however a higher score would require more intervention. Following an independent analysis of the video from each scenario, three education team members experienced in using the OSAT tool populated a scenario score for each team. Although all of the scenarios required different skills and offered different degrees of difficulty, the scores as a percentage generally improved from scenario one to scenario four. There is some variability in scores from group two at Blackpool Teaching Hospitals and group three at St. Helens & Knowsley Teaching Hospitals. At the time of writing this paper we are unsure of why the scores would decrease; however anecdotally, the variability in the latter group is reflected in the fact that only one student attended the last two scenarios.

**Table 1:** The score generated on completion of each UMUST

Scenario (in order of which they were carried out)	Scenario Score with percentage score in brackets							
	Group one St Helens & Knowsley	Group one Blackpool	Group two St Helens & Knowsley	Group two Blackpool	Group three St Helens & Knowsley	Group three Blackpool	Group four St Helens & Knowsley	Group four Blackpool
Dehydration 44 Maximum Score	24 (54 %)	20 (45 %)	14 (32 %)	27 (61 %)	20 (45 %)	21 (48 %)	15 (34 %)	16 (36 %)
Opiate Overdose 50 Maximum Score	26 (52 %)	31 (62 %)	25 (50 %)	31 (62 %)	26 (52 %)	33 (66 %)	25 (50 %)	32 (64 %)
COPD, Pneumonia 52 Maximum Score	30 (58 %)	30 (58 %)	22 (42 %)	29 (56 %)	12 (23 %)	39 (75 %)	27 (52 %)	Cancelled
Sepsis 42 Maximum Score	26 (62 %)	29 (69 %)	27 (64 %)	24 (58 %)	13 (30 %)	27 (64 %)	25 (60 %)	27 (64 %)

### Results of the questionnaire

Eighteen participants out of a possible 33 returned the questionnaires: nine were from Blackpool Teaching Hospitals and nine were from St. Helens & Knowsley Teaching Hospitals.

When asked how vividly they could remember the scenarios they were involved with in UMUST 18 trainees answered this question and 30 % said they could remember the scenarios “very vividly”, 58 % “vividly” and 12 % “quite vividly”. No respondents chose the “not at all” category. Eighteen trainees indicated that since they attended final year UMUST they had in real life been exposed to similar deteriorating patient physiology as they were required to recognise and manage within UMUST.

All 18 answered yes to the question “Do you think the UMUST programme has helped your approach to real life unplanned emergencies?” Sixteen respondents could give examples of how UMUST had helped their approach to real life unplanned medical emergencies. Eighteen trainees answered yes to the question “Was UMUST useful in preparing you to work as a Foundation Trainee?” Eighteen indicated that they found the debriefing sessions useful. Thirteen respondents gave examples of how UMUST could be improved for future cohorts of students. Table 2 summarises these responses.

**Table 2:** Summaries of free text responses from the questionnaire

Do you think the UMUST programme has helped your approach to real life unplanned emergencies? If yes, How do you think your experience of UMUST changed or helped you with the management of that situation?	Can you give examples of how UMUST has helped your approach to real life unplanned medical emergencies?	How has UMUST been useful in preparing you to work as a Foundation Trainee?	Did you find the debriefing sessions useful?	How do you think UMUST could be improved for future cohorts of students?
Number of FY1 trainees citing this in brackets/summary of comments	Number of FY1 trainees citing this in brackets/summary of comments	Number of FY1 trainees citing this in brackets/summary of comments	Number of FY1 trainees citing this in brackets/summary of comments	Number of FY1 trainees citing this in brackets/summary of comments
(5) Experience of ABCDE, UMUST scenarios when called to patients.	(7) Being more confident in ABCDE approach when starting as an FY1	(7) UMUST encouraged teamwork	(4) it was useful to learn from seeing errors	(6) More UMUST sessions
(4) Teamwork, less panic when faced with these situations.	(5) Working in a team	(4) Gives experience of ABCDE	(3) It encouraged reflection,	(2) Introduce a more multidisciplinary approach (e.g., students nurses) and shorten the de briefing
(3) Increased confidence, less panic holding a hospital pager.	(3) Gives confidence generally	(3) Gives experience of common scenarios FY1 doctors encounter	(2) It was useful to see how treatment can be streamlined, teamwork	(1) make calls to doctor more senior to us, make all students do it, give strict roles to students to stop people doing the same roles each time, have a GI bleed scenario, have 2 scenarios in a week, more input from senior clinicians, use more defibrillators, put summary of scenarios completed in the portfolio, make participants assess themselves, allow the staff to run through the first one with students, tell some wards to be more forgiving for students attending UMUST.
(2) Managing acutely ill patients, increases knowledge	(2) Gives structure when seeing patients	(2) being on call, crash bleep, provides a template for initial assessment of unwell patients		
(1) Resuscitation, just knowing what to do as an FY1, uses UMUST to deal with cardiac arrests, invaluable to practice in a safe environment, can think outside the box when seeing patients, structure to fall back on if unsure.	(1) helps with arrest calls, gives skills to stabilise patients before a senior arrives, can manage an emergency single handed, guides further learning, shows communication is vital, helps at paged calls, wearing gloves as directed by UMUST, initial assessment of unwell patient, recognises common diagnoses, similar scenarios were seen, gives a chance to go through emotions like fear and excitement.	(1)resuscitation, human factors, experience of emergency calls, communication skills, decision making, recognised limitations/know when to call for help, helps stay calm, seeing similar scenarios, gives a system to use when unsure what to do.	(1) Highlights personal strengths and weaknesses, useful to see improvements over the scenarios, can identify learning goals, good but would be better to have senior clinicians there, gives experience of being stressed.	

### Focus groups

A total of 19 students participated in the four focus groups across the two sites: 12 participants from Blackpool Teaching Hospitals and seven participants from St. Helens & Knowsley Teaching Hospitals.

#### General feedback on the programme

All focus group participants said they had enjoyed the UMUST programme and felt it was a useful experience. A number of students said that they were apprehensive about participating in the programme at first.

*“I was certainly apprehensive about doing it, having the bleep etc. watching the stuff back, nobody wanted to do it but by week two, week three we were much better and it definitely improved my confidence in such situations.”*

*“It is good practice to be honest, to be actually prepared to be a FY1 before it’s like real life patients.”*

*“The best thing about it is the practising of real life situations and you know, life threatening situations and on a mannequin is better than practicing on the first place with a real human being.”*

Those students who had no prior experience in simulation found the programme to be more difficult than those who had; but generally that was related to knowing about the model of the mannequin.

*“It is also like getting to know the model because when you turn up for the first time you don’t know what it can do.”*

A number of students had used simulation previously but for those who had no prior experience, an introductory session would have been useful. The students found the scenarios got easier as they progressed through the programme and their confidence in the skills and model improved.

*“Yes and with the model it is getting confidence in your own ability as well and the whole thing gets a lot slicker.”*

#### Holding a hospital pager

Carrying a pager was a major part of the UMUST experience for the students. On occasion it raised questions on the ward they were working on at the time.

*“The first time mine went off one of the doctors said to me where did you get that from?”*

A small number said it caused “dread” whilst others said excitement; but all of the students were relaxed about it by the end of the scenarios.

Some students forgot they were holding the pager but for others it was always at the back of their mind.

*“I think it replicates the crash bleep quite well…but especially the first one, it’s hanging over you, you feel on edge…”*

*“It gets better but for the first scenario it is permanently in your hand.”*

#### Is UMUST relevant to the final year curriculum?

All students said that the programme linked well with the rest of the final year curriculum at the University of Liverpool, particularly in that it took place after final examinations.

*“In the fourth year we are reading books or whatever or getting on the wards due to finals. However, in the fifth year you are thinking about working as an F1..so you need to be operating at a certain level of competence so I think it is definitely worth it (UMUST) in the fifth year.”*

The programme complemented Advanced Life Support training:

*“ALS is more about saying what you would do rather than whereas UMUST is about doing and getting a response from a patient.”*

Again, it was said that it was useful to have had previous experience in simulation, but UMUST was seen as including more realistic scenarios.

#### Are the scenarios relevant?

A number of students had seen some of the scenarios during their clinical attachments and they felt they were relevant.

*“I had an opiate overdose on the ward a couple of days after the scenario here and it ran so much smoother because I had just done it here and while it was happening I was like more aware of what was going on…because I had gone through it in real time I felt more comfortable than they (the FY1s) did because it was the first time they had seen it.”*

*“It’s got good scenarios and not very far from what we will be facing in the future.”*

#### Does UMUST help prepare for the first postgraduate year?

All the students felt it was good preparation for the Foundation Year.

*“As you go on you get more and more confident and get less worried about called to the things on F1”*

Also, the handover was seen as very useful by all groups:

*“That’s very useful because we don’t get enough practice on s-bar*.”

The simulation centre was genuinely felt to be a place where the students could relax and learn/practice the skills they will need post-graduation. They also said the programme reflected a real life clinical setting as there were no senior doctors involved in the scenario until they used the telephone. A positive outcome was being able to go through ABCDE in a simulated environment.

*“I think the best thing about it is and has been said before is the confidence of going there and going through ABCDE, that systematic approach so even when everything seems to be going wrong really you have something to come back to.”*

The programme was seen as very important in helping practicing team working and communication skills.

*“I think communication skills are really important because you are not going to be on your own for your F1, you are going to be working as part of a team and especially if you are on the response team you are going to work and communicate with people. “*

The handover was also seen as important.

*“It feels a bit daft when you have people watching you and you know they are behind the screen, but it is useful to practice that handover isn’t it as it isn’t something you would usually get on the rest of our clinical experience of 5th year.”*

#### How UMUST sessions are distributed throughout the year

The participants’ views on this depended on where they had been based. Participants from St. Helens & Knowsley Teaching Hospitals felt the timetabling of their programme was the most advantageous:

“*I think that having them bunched together as we do is more realistic…as an F1 you will be expected to go and see someone who is very sick once a week”*

However, participants at Blackpool Teaching Hospitals preferred the timetabling of their programme.

“*I think it works quite well because we see things on the wards and stuff over the course of the year and you can pick up more knowledge there, so you can bring it all together when you do UMUST.”*

#### Debriefing sessions

The debriefing sessions were seen as a vital element of the learning process. However, nearly all the participants found the sessions to be difficult at first.

*“I think you need the first one get over the cringe factor don’t you?”*

As they experienced more UMUST sessions, the students grew more accustomed to the debriefing process. They did feel that only one viewing of the scenario was needed; but all recognised it was essential to the UMUST experience.

*“I think the reflection itself is very good – I just don’t think you need to have the whole thing played…twice…you watch it and you spot certain things and the second time you have forgotten what you were going to say.”*

Also after a couple of sessions some students felt

“*You can predict the points they are going to stop (the video) anyway.”*

Participants felt that the debriefing sessions gradually became easier and by the end of the final session as they were watching more for what they were doing rather than how they sounded/looked on film.

#### How could the programme be improved?

Those who were unfamiliar with the mannequin would have liked a taster session to get accustomed to the model.

One person said “*It has been absolutely excellent; it is challenging which is good…it is digging out what you already know and building on your previous skill…I think everyone has something to gain from this programme and they should keep it going.”*

There were a number of suggestions for improvement, the most popular of which was to have more UMUST sessions; but it was felt that four sessions was the minimum.

*“I think within those four times you can…find mistakes…and learn from them again…by the 4th time you are more confident and the team is working well.”*

Participants suggested that handouts on the longer-term management of illnesses and current clinical guidelines on treatments could be given, whilst some suggested that the programme could be expanded to other healthcare professionals. A number of students said it would it be useful to have feedback from senior doctors about the clinical aspects of the scenario.

*“…maybe a tutorial at the end when everyone has done it, have say the anaesthetist come in at say five O’clock and talk through management of that patient.”*

Approximately half of the participants suggested that the optimum size of each cohort was three people; but this was acknowledged not to be a major downfall of the programme. As previously noted, the majority of participants felt that the duration of the debriefing sessions could be reduced and that they only needed to watch the video once.

## Discussion

Overall the UMUST programme was received positively by those who participated. They felt it was both useful and enjoyable, and they felt the scenarios were realistic. This was reflected in the feedback from the questionnaires as all participants stated they had since experienced deteriorating patient physiology and that the programme helped in their approach towards dealing with this. Furthermore, the respondents stressed the importance of learning non-technical skills as part of the programme, such as team working and effective communication; but most importantly all participants felt the programme enhanced their overall preparedness to work as an FY1 trainee.

Gaining practice and application in ABCDE was cited in feedback from both the focus groups and the questionnaires as a primary benefit of UMUST: indeed, if this in itself was the only benefit of UMUST then it would have been seen to have been a worthwhile project.

Although the programme used simulated scenarios to enact emergency situations, participants nonetheless said during the focus groups that they had experienced the same level of anxiety as they would as junior doctors faced with medical emergencies, and the results of the questionnaire indicate that the programme had helped to develop the confidence of participants in terms of holding a pager. This is an element of the programme which, arguably, made the learning as authentic as possible. Together with the experience of working in a group, this can be seen as bringing the students into a community of practice [13, 14] in terms of learning to function effectively as part of a multi-professional team. Both the questionnaires and the focus groups reinforced this notion in terms of the necessity for effective communication: the students were required to communicate with the nurse when they arrived at the simulation centre; and then again with each other at different times during the scenario; and finally they were required to enlist senior help or to ask for advice using the S-bar technique [15] in order to complete the scenario. Again, this served to enhance the authenticity of the simulation – and ultimately better preparing participants for clinical practice – by immersing them in those communities of practice which they would be required to belong to as FY1 trainees.

### Limitations of the programme

#### Number of participants

A small number of students participated in the programme and the response rate to the questionnaires was limited, partly due to the difficulty in contacting the participants once they had graduated to FY1 doctors. The limited number of responses is reflected in the small study population.

#### Feedback mechanisms

The questionnaires did not ask for the participants’ self-perception of their experience but rather asked them specific questions, which may have been limiting in terms of the diversity of feedback generated. Moreover, the questionnaires were distributed approximately half way through the FY1 year in order that participants had undergone some experience of working as doctors; but this timeframe was still relatively close to their graduation. The focus groups were held before they had graduated and worked as doctors but their reflections about UMUST during these focus groups can be triangulated with the views on the questionnaires after they had experienced working as FY1s.

#### OSAT tool

The OSAT scores reflected a general improvement in the participants’ performance across the four scenarios; however the complexity of the scenario and a reduction in the number of participants may have affected the performance scores. It is unsurprising to see that the ‘pneumonia’ and ‘sepsis’ scenarios caused some variability in the scoring as the clinical pathway for the management of both of these conditions was under review at the time of implementing the programme. Also, an inherent limitation of this tool is that it measures technical skills following a rigid framework.

#### Moving forward

A number of changes have been made to the programme following the feedback from the focus groups and questionnaires including a strengthened induction programme that orientates participants to the training programme, thereby offering them the opportunity to become familiar with the clinical skills facility and the mannequin. More teaching around the scenarios has also been implemented.

## Conclusions

UMUST has been extremely popular with the participating students who believe the programme has helped to prepare them to work as junior doctors and this study adds to ever increasing number of studies promoting the advantages of simulation based education [16]. The fact that the programme has been run across two different hospitals using slightly different means of delivery shows that it can be adapted to suit local resources. UMUST is still being run at both sites and has led to the development of other simulation experiences for students. We believe that the programme is a safe and realistic way to assist medical students in making the transition to junior doctor.

### Ethics approval and consent to participate

Ethical approval was sought and gained from the University of Liverpool Committee on Research Ethics (reference number 201106084), the Research and Development Audit Manager at St. Helens & Knowsley Teaching Hospitals NHS Trust and the Research and Development Committee at Blackpool Teaching Hospitals NHS Foundation Trust.

All participating students were issued with an information sheet that outlined the programme when they attended their general induction at the hospitals. They were required to provide informed written consent to take part in the focus groups or interviews.

### Consent for publication

Not applicable. No individual person’s data is including in this study. Written consent was gained as indicated above on the basis any quotes in the focus groups or on questionnaires could be used as long as they would be not be attributable to any individual.

### Availability of data and materials

The focus group transcriptions and interview data are saved and stored on a password protected work computer of the corresponding author SW. These have no personal identifying markers on them and are available for inspection. The original focus group audios and questionnaires have been deleted/destroyed in line with ethical approval guidelines.

## Abbreviations

ALS: Advanced Life Support
FY1: Foundation Year 1
GMC: General Medical Council
OSAT: Objective Structured Assessment Tool
PBL: Problem Based Learning
UK: United Kingdom
UMUST: Unexpected Medical Undergraduate Simulation Training
